# The Involvement of Girls and Boys with Bullying: An Analysis of Gender Differences

**DOI:** 10.3390/ijerph10126820

**Published:** 2013-12-05

**Authors:** Marta Angélica Iossi Silva, Beatriz Pereira, Denisa Mendonça, Berta Nunes, Wanderlei Abadio de Oliveira

**Affiliations:** 1 University of São Paulo at Ribeirão Preto College of Nursing, WHO Collaborating Centre for Nursing Research Development, Ribeirão Preto 14040-902, Brazil; E-Mail: wanderleio@usp.br; 2 Institute of Education, University of Minho, Braga 4710-057, Portugal; E-Mail: beatriz@ie.uminho.pt; 3 Institute of Biomedical Science Abel Salazar, University of Porto, Porto 4050-313, Portugal; E-Mail: dvmendon@icbas.up.pt; 4 Bragança Sub-Health Region, Bragança 3501-862, Portugal; E-Mail: bnunes786@gmail.com

**Keywords:** school health services, bullying, public health

## Abstract

This exploratory and cross-sectional study aimed to identify the prevalence of bullying in a group of students and analyze the data regarding the gender of those involved in the violence. A questionnaire adapted from Olweus was applied in seven elementary education schools in Portugal. The sample consisted of 387 students between 7 and 14 years old. Data are presented in terms of descriptive statistics and differences between proportions were analyzed using chi-square tests. The gender analysis of victimization and aggression shows that boys and girls are both victims and aggressors, and there are significant differences in involvement in bullying between genders and the roles played. Boys are victims more often when considering different types of bullying, although significant differences were only found for physical aggression. Strategies that include gender roles are a priority for prevention and careful attention to this phenomenon in the school context. The questions addressed contribute to a broader understanding of the phenomenon, emphasizing the differential participation of boys and girls in bullying.

## 1. Introduction

Schools are responsible for children’s wellbeing and, when this does not occur, children are deeply affected. As early as the first years of life and through play, children socialize, learn standards and develop an idea of limits. They learn by doing and feel continuous satisfaction in the search for new experiences, in which it is the school’s function to provide a space for wellbeing. In addition to personal satisfaction, this concept of “welfare” also covers effort, commitment and learning. On the other hand, some children experience times of difficulty relating with their peers and are victims of bullying. Children identify recess as a very valuable space which they love, but also as the sphere in school where bullying occurs more frequently [1]. Hence, the school institution is highly meaningful for children as a means of socialization and learning. It is through education that children have access to new, socially produced and systemized knowledge. School is also a privileged space for the promotion of quality of life in a broader focus, from the perspective of building citizenship and developing the different actors this universe comprises. 

However, research has demonstrated that, currently, school is indicated as one of the possible spaces were violence, bullying and a lack of discipline are produced and reproduced in their most different forms, as opposed to what would be expected from the school context as a space for socialization and protection. In a broad sense, the political, cultural, social and technological transformation society is going through currently also determine the forms in which violence appears in schools, especially with respect to internal and relational violence.

In school, social relations can take specific forms, such as bullying between peers, which different studies around the world have looked at, as this phenomenon is an increasing source of concern, both because of its growth and because it affects younger and younger age groups, reaching the first years of education. This turns it into an important and severe social, educational and public and mental health problem [1,2,3,4]. Specifically, bullying can be defined as aggressive behavior among peers, intentionally and ongoing and can be subdivided and characterized into three types: physical, verbal and indirect. Bullying can be characterized from three criteria: intentionality, repetition and imbalance of power [5]. It is a form of aggressive behavior, and is usually malicious, deliberate and persistent behavior between peers, which can last weeks, months or years, during which time it is difficult for victims to defend themselves [3,6].

In Portugal, as in other sociocultural contexts, it is assumed that definition is considered part of an international code. In this respect, it is important to note that unique definitions or those that have greater conceptual proximity collaborate, leading to greater accuracy and consistency between the results obtained by different researchers in the world. Culturally based studies thereby contribute to the construction of solid, cohesive knowledge concerning the investigated theme [5].

An analysis of this phenomenon in schools, according to different authors [1,7,8], reveals that children involved in bullying behavior can play different roles: (a) aggressors/intimidators; (b) victims; (c) aggressors who are also victims and (d) passive observers. These observers are neither directly involved as aggressors nor as victims. As such, they can play a number of different roles: they can defend the victims, thus reducing this type of behavior; they can support the aggressors, actively reinforcing intimidation; children who merely observe are neutral or indifferent.

The authors believe that to characterize bullying in school, gender is one of the fundamental variables in understanding this phenomenon and supports possible interventions. The gender concept seeks to distinguish between the social and historical construction of male and female on the one hand and sex on the other, as well as to explain power relationships between men and women and how they relate in and with society [9,10]. Gender constitutes a structure of social practice that establishes relations of power, attitudes and hierarchies, not only among people, but also among groups and institutions, which would simply overcome the analysis or individual perception of being female or male. This category permits an understanding of socially predefined roles for men and women as perpetrators of unequal hierarchical relations [9].

The scientific literature provides results in this direction, emphasizing issues of gender in analyses of school bullying. Research on bullying suggests that boys are more prone to be both bullies and victims of bullying, especially in its physical expression, since girls are more likely to engage in situations of indirect bullying, such as teasing or gossip about peers [7,11].

In a study that analyzed the influence of gender in the perceptions of 2,295 Spanish teenagers (54.3% boys and 45.7% girls) aged 12 to 16 years (M = 13.8, SD = 1.4) regarding roles in bullying situations, boys were revealed to interpret various forms of bullying as mechanisms of interaction between peers, while the girls recognized in the same behavior the intention of harming another and an imbalance of power [12]. A study developed with 206 high school football players between 14 and 18 years of age who attended one of three high schools in the Midwestern United States verified that issues concerning masculinity and the influence of other male figures in adherence to or approval of bullying behaviors, as well as the influential role that these players can exert positively on other boys in the sense of establishing a culture of non-violence in schools [9]. 

In a recent study using a representative sample of 1,500 Spanish students enrolled in compulsory secondary school during the 2007–2008 academic year, it was revealed that concerning bullies, boys are involved in all kinds of bullying incidents to a significantly higher degree than are girls, except in cases of indirect manifestation of the phenomenon (such as speaking ill of someone, for example) in which girls are significantly more associated with than are boys. For victims, too, the boys experience direct experiences of bullying, such as physical aggression, and girls are more involved in situations of indirect violence, such as malicious gossip [13]. Another article that reviews integrates empirical findings on the risk factors associated with bullying and peer victimization in schools, found that many studies report that boys are in general more likely to engage in bullying than girls, and boys are commonly victims and perpetrators of direct forms of bullying, while girls experience indirect bullying [14]. 

The investigations of bullying have been of the characterization of the processes involved and the intervention roles developed by different actors (students and teachers, for example). These studies are justified by the increased prevalence of occurrences of the phenomenon in schools and the impact they have on the lives and health of children and teens. Specifically, on the prevalence of bullying in the locale studied, a study conducted in northern Portugal with a sample of 360 public school students (53.3% boys and 46.7% girls, with an average age of 12.36 years) identified an average rate of involvement in bullying situations in schools at 27.5% of students [15]. Another study, conducted in the north and south of the country with 4,092 students, aged between 10 and 16 years (53.1% boys and 46.9% girls), verified that in the north the rate of students who reported having bullied other students was 21.6% and 15.4 had intimidated their fellow students. In the South, 19.3% reported being bullied and 16.0 practiced bullying [16]. These high rates of the occurrence of bullying in children’s and teens’ lives can contribute to the development of physical and emotional problems, particularly stress and a risk of decreased self-esteem. They can also develop anxiety and depression, feel unhappy and, in more severe cases, even develop suicidal ideas [3,17].

Studies have shown that contextual and relational aspects, as well as gender, need to be valued in proposing actions to combat the problem and justify research that advances investigation of the phenomenon. In this way, the discussions presented concentrate on a change of focus in relation to other studies that address bullying as something that arises from the characteristics of individual students, but ignores the involvement in terms of gender relations and their interconnection with other elements of culture and the contexts in which the phenomenon evolves. Thus, Portugal while has research on bullying, contextual approaches are still needed, as well as observation of other variables in order to understand the dynamics of the phenomenon. This is one of the major contributions of this study.

In view of the above, the intent of this study was to answer the following questions: What is the prevalence of bullying among children by gender in the study region? What characterizes boys’ and girls’ behavior of bullying others and being bullied? Who do the children ask for help in situations of victimization? This study is designed to identify the prevalence of bullying in a group of students and analyze the data regarding gender for those involved in the violence, as well as to determine from whom they request help in situations of victimization, as support networks are fundamental to outlining intervention programs. 

## 2. Methodology

### 2.1. Study Area and Data

The study was carried out in 2010, in Bragança, located in the Sub-Region of Alto Trás-os Montes, Northern Portugal, an impoverished interior area with a high level of emigration from villages to the towns in the same region or on the littoral zone. 

This is an exploratory and cross-sectional study, developed in seven elementary education institutions that are part of the public school system of Portugal, and all students enrolled in these schools from the second to the sixth year of primary school were invited to participate in the research. The final convenience sample included 387 students and consisted of 195 (50.9%) girls and 188 (49.1%) boys; four students did not answer this question.

The study participants were between 7 and 14 years old, with 88.1% of the sample concentrated in the group between 8 and 12 years of age. The mean age was 9.9 years (standard deviation 1.6), corresponding to 9.8 years (standard deviation 1.6) for boys and 9.9 (standard deviation 1.7) for girls.

The data collection of was accomplished by teachers, previously trained, having applied the questionnaire in the classroom. In each room, the questionnaire administration procedure lasted approximately 40 min.

### 2.2. Instrument to Collect Data

The questionnaire was adapted from Olwues’ questionnaire [6], validated for a Portuguese population in a previous study and organized in sections [1]. The first was related to socioeconomic data, the second to the identification of victimization behaviors, the third to aggression and the fourth to friendship by the nomination of peers and the children’s perception of school recess. The students answered the questionnaire during a normal class period. Teachers with proper training for this purpose applied the questionnaire. Data are presented in terms of descriptive statistics with means (standard deviations) for continuous variables and percentages for categorical variables, such as for categories of victimization and aggression. Differences between proportions were analyzed using the Chi-square tests. The statistical significance level was set at α = 0.05. The collected data were processed electronically and analyzed using the Statistical Package for the Social Sciences^®^ (SPSS, SPSS Inc., Chicago, IL, USA) version 17.0. 

The victimization variable was recoded into two levels, non-bullied and being bullied, including all being-bullied cases, that is, being bullied “1 or 2 times”, “3 or 4 times” and “5 or more times” during that period. Similar recoding was adopted for the aggression variable.

In this phase, considering that much of the bibliography refers to bullying behavior as continuing aggression, despite the lack of a consensus in research about the duration of the behavior in order to be characterized as bullying, the victim variable was further recoded as: (i) “Non-victim” for children who answered they had never been bullied or bullied only once or twice during that period and (ii) “Victims” for children who had been bullied “three times or more” during that period. A similar procedure was performed for the question concerning aggression.

### 2.3. The Ethical Issues

In regard to ethical issues, this study was authorized and approved by the Ministry of Education of Portugal, Direcção-Geral de Inovação e de Desenvolvimento Curricular (DGIDC), according to the survey No. 0163700001, filed on 18 November 2010. Students were invited to participate in the study and, if accepted, their guardians provided written consent to participate.

## 3. Results

Regarding the process of victimization, 378 students responded to two specific questions, and 53.2% had never been bullied. In total, 46.8% children indicated they had been bullied during the school period analyzed: 22.8% once or twice and 24.1% three or more times (Table 1/Table S1 in the supplementary file).

**Table 1:** Percentage of children who reported being bullied according to gender.

Group	Boys			Girls			Total	
	*n*	%		*n*	%		*n*	%
Were not victims	83	45.1		118	60.8		201	53.2
Were bullied 1 or 2 times	45	24.5		41	21.1		86	22.8
Three or 4 times	19	10.3		19	9.8		38	10.1
Five or more times	37	20.1		16	8.2		53	14.0
Total	184	100.0		194	100.0		378	100.0

The analysis of victimization levels according to gender reveals a statistically significant difference in behavior patterns (X^2^ = 14.35, *p* = 0.002). Bullying victimization levels are higher for boys, mainly when considering more frequent victimization situations (five or more times), with 20.1% for boys and 8.2% for girls. The gender analysis of victimization and aggression shows that both boys and girls are victims and aggressors, although the percentages of being bullied and bullying others are significantly higher for boys (Table 2). 

**Table 2:** Percentage of children who reported being bullied or bullying others according to gender.

	Boys			Girls			Total		*Chi-Square*	*p*
	*n*	%		*n*	%		*n*	%		
Being bullied									9.37	0.002
no	83	45.1		118	60.8		201	53.2		
yes	101	54.9		76	39.2		177	46.8		
Bullying others									9.84	0.002
no	100	54.6		137	70.3		237	62.7		
yes	83	45.4		58	29.7		141	37.3		

The analysis of the question of victimization in combination with the question of aggression reveals that a group of victims, a group of aggressors and yet another of children who are both victims and aggressors exists. In this sense, 267 (70.6%) were neither victims nor aggressors. Seventy (18.5%) were victims but not aggressors; 22 (5.8%) were aggressors but not victims and 19 (5.0%) were both victims and aggressors. This approach seems to give the best evidence of a profile likely to lead to a better understanding of the problem (Table 3).

The comparison between percentages of child victims and aggressors, according to the results reported in Table 2, reveals a difference of 9.5 between the percentages of being a victim and being an aggressor (46.8% *vs*. 37.3%). When comparing these percentages according to the stricter criteria used in Table 3, *i.e*., continuing bullying behavior, this difference between victims and aggressors increases to 12.7 (23.5% for victimization and 10.8% for aggression). 

**Table 3:** Percentage of children who were being bullied, bullied others or both.

Group	Boys			Girls			Total	
	*n*	%		*N*	%		*n*	%
Neither being bullied nor bullying others	112	62.2%		151	77.8%		267	70.6%
Being bullied	38	21.1%		32	16.5%		70	18.5%
Bullying others	14	7.8%		8	4.1%		22	5.8%
Being bullied and bullying others	16	8.9%		3	1.5%		19	5.0%
Total	180	100%		194	100%		378	100%

Note: Missing values n = 4 (gender differences: chi-square = 16.33, *p* < 0.001).

The analysis of the type of aggression the children were victims of, shows that 14 of the 387 respondents did not answer these questions. In 106 cases (28.6%), the children were victims of physical aggression (hits, punches and kicks), while 76 (20.5%) were victims of theft (their belongings were taken away) (Table 4/Table S2 in the supplementary file). 

**Table 4:** Percentage of being bullied typology, according to gender.

Being bullied typology	Total			Female			Male		p *
	*n*	%		*n*	%		*n*	%	
Hitting, punching and kicking	106	28.6		40	21.3		66	36.3	*p* < 0.001
Steal, taken belongings	76	20.5		37	19.7		39	21.4	NS
Threatened	60	16.2		28	14.9		32	17.6	NS
Insulting	134	36.2		60	31.9		74	40.7	NS
Rumors spread	73	19.7		41	21.8		32	17.6	NS
No one talks to her/him	28	7.6		11	5.9		17	9.3	NS
Cyber bullying	4	1.1		3	1.6		1	0.5	NS ^(a)^
Insulted due to color or ethnic origin	13	3.5		6	3.2		7	3.8	NS
Other forms of victimization	18	4.8		12	6.4		6	3.3	NS

* *p*-value; NS—Not significant (*p* > 0.05); ^(a)^ Fisher exact test; Note: Exceeds 100%, since children could check more than one response.

In both cases, practices are more frequent among boys than among girls, with significant differences for physical aggression (hitting, punching and kicking) (X^2^ = 10.16, *p* < 0.001). For other victimization subtypes, gender differences were not statistically significant, although higher percentages of boy victims are observed in all cases, except for “spreading rumors” and “disseminating images or messages by mobile phone or internet” (cyber-bullying) with a view to damaging the classmate’s image and “other forms of victimization” which has a higher percentage of girl victims. 

Although most of the children and adolescents victims of bullying sought multiple sources of help, having talked about this experience with their families (parents, siblings), at school (teachers, employees), in their peer group (friends), 16% of the victims did not tell anyone (Table 5). It should be highlighted that the percentage of students who did not tell anyone they were victims is particularly important, because it represents a form of hiding the occurrence of bullying, thus constituting a risk group. Gender differences were also found to reveal a significantly higher percentage among girls (21.9% *vs*. 10.9% (X^2^ = 3.93, *p* < 0.05)). In general, the results indicate that boys report the occurrence of bullying more often to friends, parents, brothers and teachers (Table 5/Table S3 in the supplementary file).

**Table 5:** Whom children inform when they were bullied, according to gender.

Who did you tell?	Total		Female		Male		p *
	*n*	%	*n*	%	*n*	%	
Did not tell anyone	27	15.5	16	21.9	11	10.9	*p* < 0.05
Told one or two friends	54	32.0	22	30.1	32	31.7	NS
Told friends	44	25.3	15	20.5	29	28.7	NS
Told the teacher or class director	75	43.1	29	39.7	46	45.5	NS
Told the father or tutor	86	49.4	33	45.2	53	52.5	NS
Told a brother or sister	28	16.1	10	13.7	18	17.8	NS
Told an employee	55	31.7	25	34.2	30	29.7	NS

* *p*–value; NS—Not significant (*p* > 0.05); Note. Exceeds 100%, since children could check more than one response; Missing values n = 3.

## 4. Discussion

The percentage of victims exceeds that of aggressors, and this difference increases when considering continuing situations of bullying. Gender differences in the subtypes of bullying play different roles in the pupils’ health [18]. Boys are victims more often when considering different types of bullying as a whole, although differences are not always significant. Significant differences were found for physical aggression and insults (name-calling) when comparing boys with girls, in line with earlier research [3,6,18]. Insults as a more frequent practice among children this age also confirms earlier studies, although differences are less frequent at the level of gender [3]. The most frequent forms of victimization are insults, with one in every three children being a victim of insults, followed by physical aggression, with one in every four. Hence, using physical aggression and insults can be considered a more evident profile for boys. Despite the lack of significant differences, less assumed and more indirect forms of aggression seem to be more frequent among girls (talking about the other person and other forms of victimization), although percentages are low. The data converge with those found by other studies, which did not identify significant differences in involvement in bullying between genders and the roles played (bullies or victims) [19]. 

A review of studies on victimization among adolescents and gender issues identified strong evidence that bullying is directly related to issues of sexuality and gender, as these are associated with other variables such as feelings of belonging to a social group and school [20].

Analyses of the role of gender in the involvement of children and adolescents with bullying reveal the existence of significant similarities with regard to the frequency and modalities of participation in the situations of the phenomenon. In the sample group, the boys engage more in both forms of bullying (direct and indirect) and the scientific literature finds that the indirect expression of violence in its verbal form is most frequently present in the relationships between girls, while boys are involved more directly in the phenomenon (physical violence) [7,12].

A study of Spanish adolescents verified that stereotypical characteristics of masculinity are strongly related to the perpetration of bullying and violence in relation to both sexes, whereas feminine characteristics are negatively related to the maintenance and perpetration of bullying. This study, in contrast, found that feminine traits are more related to victimization than are male students [18].

One suggested explanation is that the stereotypical participation of boys and girls in situations of bullying has social roots, because traditionally the more aggressive behavior and violence of boys are reinforced, whereas indirect involvement or further victimization of girls is more consistent with traditional stereotypes of femininity. These stereotypes reveal the strategies used by different genders to ensure a prominent place in the group and in peer relations [13].

Understanding the importance of gender differences in bullying situations is especially important to observing aspects that contribute to the perpetuation of violence, as in the case of males, whose demands and social issues contribute to the definition of relationships based on imbalances of power and intimidation. These gender differences reflect expected symbolic content and sexual roles that are legitimized by the socialization process, which departs from a male-chauvinist model and is reproduced in school based on the social and family context girls and boys experience [9,10]. 

Gender relations manifested in people’s daily lives, emphasizing manifestations in the school context in this research, are closely linked with subjects’ social lives, especially in power relationships between genders, and between female and male representations of moral violence, especially with respect to the perceived action(s) expected from the opposite sex in these situations; this is a strong relationship of power and influence with the male gender in actions of moral violence inside schools [21]. At the same time, a study with data generated in a collective biography workshop, which sought to develop new forms of thinking about bullying, identified that a critical approach to the issue requires consideration of the normative practices in and regulatory power of schools [22]. Therefore, we must consider that the discourses and power relations that constitute the people and their ways of relating reproduce stereotyped conceptions of gender that are directly related to the structural context of society, in a logic of maintaining or preserving power relationships.

In this sense, male and female students have been guided how to handle conflict situations and in regard to the need to reconsider sexist attitudes, which strengthen socially and historically constructed gender stereotypes in school education. In this research, most pupils who were victims of bullying between peers in school mentioned they had talked to somebody about the subject, supporting results from other studies [2,21,23]. They sought more help from friends than from parents, tutors, teachers or other persons from the school itself. The same pattern was observed by other authors, which suggests that children and adolescents still consider friends and family as a space for protection, and the possibility of this type of violence as something that should be dealt with in the private sphere [1].

The debate about gender also allows problematizing the situation of victims of bullying, which often do not manifest themselves at the time of the occurrence. This may be related to the fact that they believe that this will not be of any help to them, that they are afraid of retaliation, that they will get less family support or that they consider these actions and their aggressors as hardly relevant [2]; these all constitute a movement of reproducing the social relations of gender. However, talking to someone about having been a victim reduces the probability of subsequent events. In this sense, it is essential to encourage all children and adolescents to communicate and to create the necessary conditions for them to develop supportive and sensitive behavior toward aggressions committed against themselves or others, stimulating them to tell adults, either in school, in the family or others they trust [2].

The results discussed regarding involvement in bullying situations and gender considerations allow the formation of conceptions of intervention practices in schools with a multidisciplinary and intersectorial focus. The results verify that bullying is an expanding phenomenon that deserves great attention from researchers, teachers, school boards, education and health professionals and families.

The authors believe yet it is important to attract teachers’ attention to child victims, whereas this study identified in their sample higher prevalence of victims in relation to aggressors, which also justifies its approach, creating support inside the class through students who observe the situations but do not know how to intervene, so that the latter contribute to creating a climate where bullying behavior is perceived as negative and, therefore, is not tolerated in their classes. The victims are subjects suffering the intentionality of bullying and can register this type of social interaction negatively in terms of gender relations.

## 5. Conclusions

Bullying in the school space can be explained by a set of associated factors, especially social roles, among which the climate created in school stands out, which is not favorable to gaining cooperation regarding bullying. A child’s education in the family is greatly centered on competitiveness and victory at any price, but is not directed to the acquisition of gradual competencies, according to age and achieved through cooperation and solidarity. 

This study shows that bullying is still prevalent in the male gender, manifesting itself through physical assault and insults. Strategies should be implemented that include gender relations as a priority for prevention and care of bullying in the school context.

In their work process, areas like health, education and social work as social practices should establish the care-giving dimension from the perspective of promoting individual and group quality of life, in response to a plural dimension of needs and requests that find interdisciplinary practice to be a privileged space to construct an intersectoral, articulated and egalitarian care and intervention model that can broaden effective and coherent work regarding bullying.

It is worth mentioning that the approach of this study contributes to a broader understanding of the phenomenon, inasmuch as it provides data on the variables of gender involved in bullying and sheds light on the complex issues of the problem. It becomes clear that the establishment of nexuses that explain bullying and that establish causal relations broaden the focus of the research approach and leads to the embrace of other variables that are present in different cultures and manifested also in different ways, like gender. Undertaking a population-based study in the near future is important for understanding the complexity of this phenomenon that causes suffering in many children and teens in the school environment. Although not responding to the global problem, the study does thicken the contributions to the establishment of an explanatory model of bullying taking into consideration the cultural reality of a country in the European south-west, and trends that the phenomenon takes on. This triggers progress in science and in how we face the question, during a time in which the problem is widely disseminated, and efforts are consolidated on a theoretical problem. Studies like the one presented deepen analysis of specific aspects that are essential to the understanding of the whole, from the local vantage point, which is justified, is set contributes for the dynamics of bullying and what differences it assumes in its manifestation between boys and girls.

Although this study does offer contributions to the subject of bullying, there are aspects of the study design that limit the conclusions that need to be considered. The cross-sectional design of the study precludes inferences about causality and the direction of the influence of gender as a significant predictor of bullying. Future researches with other designs can provide a more accurate picture of the differences in involvement in bullying according to sex.

## Supplementary Files

Supplementary File 1 Supplementary Information (DOC, 72 KB)
