# Supplementary material for: The Involvement of Girls and Boys with Bullying: An Analysis of Gender Differences
Source: Int J Environ Res Public Health. 2013 Dec 5;10(12):6820–31. doi: 10.3390/ijerph10126820 (PMC3881143; doi:10.3390/ijerph10126820)
Supplement: Supplementary File 1 — Supplementary Information (DOC, 72 KB) [file ijerph-10-06820-s001.doc]

*Supplementary Information*

**The Involvement of Girls and Boys with Bullying: An Analysis of Gender Differences**

**Table S1.** Percentage of children who reported being bullied according to gender.

|  | **Boys** | |  | **Girls** | |  | **Total** | |
| --- | --- | --- | --- | --- | --- | --- | --- | --- |
|  | ***n*** | **%**  ***(std adjusted residuals)*** |  | ***n*** | **%**  ***(std adjusted residuals)*** |  | ***n*** | **%** |
| Were not victims | 83 | 45.1  (−3.1) |  | 118 | 60.8  (3.1) |  | 201 | 53.2 |
| Were bullied 1 or 2 times | 45 | 24.5  (0.8) |  | 41 | 21.1  (−0.8) |  | 86 | 22.8 |
| Three or 4 times | 19 | 10.3  (0.2) |  | 19 | 9.8  (−0.2) |  | 38 | 10.1 |
| Five or more times | 37 | 20.1  (3.3) |  | 16 | 8.2  (−3.3) |  | 53 | 14.0 |
| Total | 184 | 100.0 |  | 194 | 100.0 |  | 378 | 100.0 |

**Table S2.** Percentage of being bullied typology, according to gender.

| **Being Bullied Typology** | **Total** | |  | **Female** | |  | **Male** | | | ***Chi-Square*** | | ***p **** | |
| --- | --- | --- | --- | --- | --- | --- | --- | --- | --- | --- | --- | --- | --- |
|  | ***n*** | **%** |  | ***n*** | **%** |  | ***n*** | **%** | |  | |  |  |
| Hitting, punching and kicking | 106 | 28.6 |  | 40 | 21.3 |  | 66 | 36.3 | 10.16 | | | | p < 0.001 |
| Steal, taken belongings | 76 | 20.5 |  | 37 | 19.7 |  | 39 | 21.4 | | 0.17 | | 0.677 (NS) | |
| Threatened | 60 | 16.2 |  | 28 | 14.9 |  | 32 | 17.6 | | 0.49 | | 0.483 (NS) | |
| Insulting | 134 | 36.2 |  | 60 | 31.9 |  | 74 | 40.7 | 3.06 | | | 0.080 (NS) | |
| Rumors spread | 73 | 19.7 |  | 41 | 21.8 |  | 32 | 17.6 | | 1.04 | | 0.307 (NS) | |
| No one talks to her/him | 28 | 7.6 |  | 11 | 5.9 |  | 17 | 9.3 | | 1.61 | | 0.205 (NS) | |
| Cyber bullying | 4 | 1.1 |  | 3 | 1.6 |  | 1 | 0.5 | |  | | 0.623 **^a^** (NS) | |
|  |  |  |  |  |  |  |  |  | |  | |  | |
| Other forms of victimization | 18 | 4.8 |  | 12 | 6.4 |  | 6 | 3.3 | | | 0.117 | 0.732 | |

Notes: ***** *p*-value; NS—Not significant (*p* > 0.05); **^a^**Fisher exact test; Exceeds 100%, since children could check more than one response.

**Table S3.** Whom children inform when they were bullied, according to gender.

| **Who did you tell?** | **Total** | | **Female** | | **Male** | | ***Chi-square*** | | ***p**** |
| --- | --- | --- | --- | --- | --- | --- | --- | --- | --- |
|  | ***n*** | **%** | ***n*** | **%** | ***n*** | **%** |  |  |  |
| Did not tell anyone | 27 | 15.5 | 16 | 21.9 | 11 | 10.9 | 3.93 | | *p* < 0.05 |
| Told one or two friends | 54 | 32.0 | 22 | 30.1 | 32 | 31.7 | 0.047 | | 0.828 (NS) |
| Told friends | 44 | 25.3 | 15 | 20.5 | 29 | 28.7 | 1.495 | | 0.221 (NS) |
| Told the teacher or class director | 75 | 43.1 | 29 | 39.7 | 46 | 45.5 | 0.585 | 0.444 (NS) | |
| Told the father or tutor | 86 | 49.4 | 33 | 45.2 | 53 | 52.5 | 0.896 | | 0.344 (NS) |
| Told a brother or sister | 28 | 16.1 | 10 | 13.7 | 18 | 17.8 | 0.534 | | 0.465 (NS) |
| Told an employee | 55 | 31.7 | 25 | 34.2 | 30 | 29.7 | 0.405 | | 0.525 (NS) |

Notes: ***** *p*–value; NS—Not significant (*p* > 0.05); Exceeds 100%, since children could check more than one response; Missing values n = 3.
